# Editorial: Cereals and cereal products: nutritional and physicochemical characterization and novel foods

**DOI:** 10.3389/fnut.2024.1446657

**Published:** 2024-07-01

**Authors:** Roberta Foligni, Virginia Teresa Glicerina, Agnieszka Orkusz, Cinzia Mannozzi

**Affiliations:** ^1^Department of Agricultural, Food and Environmental Sciences (D3A), Università Politecnica delle Marche, Ancona, Italy; ^2^Department of Agriculture, Forest and Food Sciences, University of Turin, Grugliasco, Italy; ^3^Department of Biotechnology and Food Analysis, Wroclaw University of Economics and Business, Wrocław, Poland; ^4^Chemistry Interdisciplinary Project (CHIP), School of Pharmacy, University of Camerino, Camerino, Italy

**Keywords:** cereals, whole grains, bioactive compounds, chemical structure, biological activity, functional foods, grain quality, edible insect

Cereals represent a fundamental component in the human and animal diet all over the world, contributing to the supply of macroconstituents such as carbohydrates, proteins and fats and microconstituents such as minerals and vitamins, fiber, and bioactive compounds with a positive effect on human health.

The qualitative aspects in terms of nutritional and physico-chemical features are essential for the characterization of the raw material and the derived products. Detecting specific analytes as indicators of quality or authenticity can help throughout the production chain (1–3). The fat fraction can be investigated with a multivariate approach to discriminate intra-varietal and intra-species differences and therefore provide important information to the final consumer as well as to the actors in the supply chain. Obtaining products with well-defined nutritional and rheological characteristics has been a challenge of recent years. To achieve this objective, new ingredients (4–7) and new technologies are included in the product formulation such as edible insects or their derived products to understand and evaluate their contribution, in terms of nutritional compounds, as well as possible alterations and final performances (8, 9). Even the bioactive compounds (for example, phenolic acids, flavonoids, tannins, carotenoids, and saponins) have been identified and characterized in the different cereal seeds and in the new designed to create a food products with increased valuable compounds extractable from food waste and by-products. Therefore, the versatility of cereals as a raw starting material opens up numerous aspects of research from the purely nutritional and physical-chemical characterization considering as well the effects on human health taking into account the application of emerging technologies and the use of innovative ingredients. Bartkiene et al., investigate the effects of different amount of psyllium husk gel on different quality characteristics of wheat bread such as porosity, specific volume, mass loss after baking, shape crust, crumb, color parameters, bread crumb hardness during storage, saccharides, and acrylamide content were evaluated. The acceptability of whole meal bread was improved, and the quantity and softness of the crumb also increased regardless of the percentage of psyllium husk gel added. The improvements were already evident by adding 5% of psyllium husk gel. The greater porosity obtained did not lead to an increase in staling with consequent extension of the shelf-life of the product. The use of psyllium husk gel has also significantly reduced the production of acrylamide, a carcinogenic and neurotoxic compound, providing added value also from the health point of view. Sanders et al., evaluated the correlation between consumption of Ready-To-Eat Cereal (RTEC) and intake by Canadian children and adults as a function of income. The analysis used data from the 2015 Canadian Community Health Survey (CCHS)—Nutrition, encompassing dietary recalls from 6,181 children aged 2–18 and 13,908 adults aged 19 and above. The researchers evaluated diet quality by employing a modified version of the Nutrient-Rich Food Index (NRF) 9.3 and categorized income levels into low, middle, and high brackets based on household size. Through multivariate linear regression analyses, the study investigated the connections between RTEC consumption, nutrient intake, and diet quality while adjusting for energy, age, and gender.

The results showed that both children and adults who consumed RTEC had higher nutrient levels, even for deficient nutrients such as calcium, dietary fiber, iron, magnesium, and vitamin D. Furthermore, RTECs provided < 10% of energy intake, < 4% of saturated fat intake, and < 9% of total sugar intake regardless of age and different income levels, also providing a third of daily intake of iron and at least 10% of the daily intake of dietary fiber, thiamine, folic acid and vitamin B6. Based on the results, the researchers concluded that RTEC consumption was linked to enhanced nutrient intake and diet quality among Canadian adults and children, regardless of household income status. They proposed that RTEC could represent a valuable and cost-effective dietary option to enhance diet quality, particularly for individuals residing in low-income households. This underscores the potential significance of nutrient-rich and affordable food choices such as RTEC in public health nutrition strategies designed to elevate diet quality and tackle nutritional deficiencies across different socioeconomic segments. Quinoa (Chenopodium quinoa) is a whole-grain with nutritional and medical values. Huang et al., in their research investigated the effects on the intestinal flora of the numerous compounds that make up quinoa and the potential applications of quinoa in enhancing the composition and activity of intestinal flora.

Bioactive substances characterizing quinoa, oxalate, tannin, phytic acid, phytosterol, phenolic acid, flavonoid, polyphenol, and total saponins (polysaccharides, bioactive peptides, and dietary fiber) were considered and evaluated. Numerous studies have been considered to show the beneficial effects on human health. In particular, most of the research, 261, demonstrates the antioxidant effect of quinoa's bioactive compounds but there are also numerous studies for the bacteriostatic, anti-carcinogenic, anti-obesity and anti-inflammatory properties. The different characteristics depending even on the different cultivars (white, red, and black). Factors that can influence the performance of the glycemic index, such as raw materials and process parameters, were investigated by Temkov et al., in correlation with improved hunger satisfaction and maintenance of satiety. The effect on physical characteristics, sensory qualities, glycemic index and appetite sensations in naturally leavened soft wheat bread were evaluated as a function of different combinations of unrefined soft wheat flours or the addition of whole grains in the formulation and alternating cooking times. Authors considered three types of commercial pre-baked frozen sourdough bread, baked to the final baking for two different times. The evaluation was carried out considering the following “appetite” descriptors: desire to eat, hunger, fullness, satisfaction, and appetite; on a 10 cm visual analog scale (VAS) scale in a course of 180 min. Furthermore, acceptability was evaluated through different sensory attributes. The physical, structural and sensorial characteristics of the products were significantly influenced by the different bread formulations (unrefined wheat flour, cereal flour or whole cereals) and by the different cooking times. The glycemic index highlighted no significant changes in sourdough bread with added cereals, while liking score and incremental area under the curve (iAUC) of satiety and satiation showed higher differences between the different formulations.

## Author contributions

RF: Supervision, Writing – original draft, Writing – review & editing. VG: Supervision, Writing – original draft, Writing – review & editing. AO: Supervision, Writing – original draft, Writing – review & editing. CM: Supervision, Writing – original draft, Writing – review & editing.

## References

[1] Tavoletti S Pasquini M Mozzon M Servadio D Merletti A Mannozzi C. Discrimination among varieties of Triticum turgidum subspecies (*dicoccon, turanicum and durum*) based on the fatty acid profile. J Cereal Sci. (2021) 99:103213. 10.1016/j.jcs.2021.103213

[2] Tavoletti S Foligni R Mozzon M Pasquini M. Comparison between fatty acid profiles of old and modern varieties of T turgidum and T aestivum: a case study in central Italy. J Cereal Sci. (2018) 82:198–205. 10.1016/j.jcs.2018.06.012

[3] Tavoletti S Pasquini M Mozzon M Foligni R. Effect of sowing season on fatty acid profile ability to discriminate modern and old varieties of common wheat (*Triticum aestivum* L. subsp. *Aestivum*). J Cereal Sci. (2024) 116:10389. 10.1016/j.jcs.2024.103864

[4] Canali C Balestra F Glicerina V Pasini F Caboni MF Romani S. Influence of different baking powders on physico-chemical, sensory and volatile compounds in biscuits and their impact on textural modifications during soaking. J Food Sci. Technol. (2020) 57:3864–38731. 10.1007/s13197-020-04418-1 32904002 PMC7447690

[5] Glicerina V Balestra F Capozzi F Dalla Rosa M Romani S. Influence of the addition of soy product and wheat fiber on rheological, textural, and other quality characteristics of pizza. J Texture Stud. (2018) 49:415–23. 10.1111/jtxs.12311 29148052

[6] Rossi S Parrotta L Gottardi D Glicerina VT Del Duca S Dalla Rosa M. Unravelling the potential of cricket-based hydrolysed sourdough on the quality of an innovative bakery product. J Insects Food Feed. (2022) 8:921–35. 10.3920/JIFF2021.018429510743

[7] Mozzon M Foligni R Mannozzi C Zamporlini F Raffaelli N Aquilanti L. Clotting properties of Onopordum tauricum (willd) aqueous extract in milk of different species. Foods. (2020) 9:692. 10.3390/foods9060692 32471174 PMC7353650

[8] Foligni R Mannozzi C Ismaiel L Capelli F Laurita R Tappi S. Impact of cold atmospheric plasma (cap) treatments on the oxidation of pistachio kernel lipids. Foods. (2022) 11:411. 10.3390/foods11030419 35159569 PMC8834114

[9] Longo E Morozova K Monica R Tundi R Savini S Foligni R. High resolution mass approach to characterize refrigerated black truffles stored under different storage atmospheres. Food Res. Int. (2017) 102:526–35. 10.1016/j.foodres.2017.09.025 29195982

